# Medial open-wedge high tibial osteotomy for the treatment of degenerative varus knee osteoarthritis in geriatric patients: a retrospective study

**DOI:** 10.1038/s41598-023-44051-4

**Published:** 2023-10-06

**Authors:** Jia Li, Feng Zhao, Wei Dong, Xiaoguang Yu, Chaohua Zhu, Sen Liu, Guoxing Jia, Guobin Liu

**Affiliations:** Department of Orthopedic Surgery, The First Hospital of Hebei Medical University, No. 89 Donggang Road, Shijiazhuang, 050031 Hebei People’s Republic of China

**Keywords:** Outcomes research, Geriatrics, Reconstruction

## Abstract

HTO has proven to be a cost-effective surgical procedure in the treatment of KOA, but few investigations have studied radiological changes and clinical effectiveness of OWHTO in geriatric patients. 76 patients were recruited in this retrospective study. According to the age, patients were divided into two groups (≤ 60, Group “Young”; > 60, Group “Geriatric”). Demographic data, radiological imaging and postoperative complications were analyzed. Kellgren–Lawrence grade (K–L), weight-bearing line ratio (WBLR); posterior tibial slope angle (PTS); American knee score (AKS); Western Ontario and McMaster Universities Arthritis Index (WOMAC) and visual analog scale (VAS) were introduced to estimate the clinical outcome of OWHTO. There were 18 male and 58 female patients in the present study with a mean age of 58.5 ± 9.2 years (ranges from 40 to 82 years); the average age was 51.4 ± 4.1 years and 67.3 ± 4.9 years for group Y and G respectively, 44.7% and 31.5% patients were older than 60 and 65 years. BMI for the 76 patients was 26.6 ± 3.2 kg/m^2^, and geriatric patients were more likely accompanied by one or more comorbidities (70.6 vs. 45.2%). There were 34 and 42 patients in group Geriatric and group Young respectively, and no significant difference of MPTA, WBLR, PTS and WOMAC, VAS, AKS and ROM between the two group (P > 0.05) were found. After more than a two-year follow-up period, postoperative WBLR, AKS, WOMAC and VAS were much more desired than preoperative, and no significant difference of these variables between the young and geriatric group (P > 0.05), however, elderly patients were more likely to suffer from a longer bone union time. OWHTO can avoid geriatric patients from undergoing secondary knee surgery in the short term, however the survival rate of OWHTO in geriatric patients should be ultimately clarified by different studies.

## Introduction

Knee osteoarthritis (KOA), a degenerative joint disease, is a well-known cause of pain and disability in daily life, and also contributes to a significant financial burden to patients, families, and society. The epidemiological investigation demonstrated that the prevalence of symptomatic KOA in patients older than 60 years ranges from 10.0 to 16.0%, and 35.0 to 50.0% for symptomatic and radiographic KOA^1–3^ respectively. Approximately 250 million people suffer from KOA worldwide, and in China, the estimated number of individuals diagnosed with symptomatic KOA reached 37.35 million^4^.

High tibial osteotomy (HTO) was first introduced by Coventry in 1965^5^, with more than 50 years of clinical application, HTO has evolved into an accurate and effective minimally invasive surgical technique. The biomechanical mechanism of HTO is to transfer the weight-bearing alignment from the medial compartment where aseptic inflammation and articular cartilage wear occurred to the relatively healthy lateral compartment, subsequently alleviating the symptoms of arthritis, and delaying or avoiding knee replacement. HTO is indicated in active patients with varus degenerative KOA and deformity dominated by tibia. Indications of HTO were discussed in the published literature, mainly including medial proximal tibial angle (MPTA) < 85, meniscus injury, partial cartilage wear that investigated by arthroscopy or MRI, and absence of clinical pain in the remaining compartments; meanwhile, age, BMI, and some other factors were also influenced outcome of HTO. Survivorship of HTO has been reported to be approximately 80–90% at 5 years and 70–80% at 10 years^6^.

Currently, HTO is mainly performed by methods of lateral closing wedge HTO (CWHTO), medial opening wedge HTO (OWHTO). CWHTO requires rich surgery experience and skillful operation technology, currently incidence of complication following CWHTO was reported range from 10 to 20%. These complications included common peroneal nerve and anterior tibial artery injury, osteofascial compartment syndrome and secondary fractures. OWHTO is characterized by postoperative ideal and intraoperative modifiable alignment, and currently, a combination of OWHTO and locking plate has become a predominant surgical procedure in clinical practice. However, the location of the aimed weight-bearing axis is still controversial. If varus deformity is insufficiently corrected and the alignment is not shifted outwardly, compressive loads of medical compartment will not effectively redistribute; meanwhile, overcorrection always leads to an accelerated degeneration of the lateral compartment and patellofemoral joint. Therefore, total knee arthroplasty (TKA) and unicompartmental knee arthroplasty (UKA) remain the gold standard indication in patients with end-stage degenerative KOA which affects multiple or single compartments of the knee.

Some scholars have demonstrated good patient-reported outcomes and satisfactory improvement of activity after OWHTO in “young” patients, but few investigations have studied radiological changes and clinical effectiveness of knee joints after osteotomies in geriatric ones. Survivorship of TKA or UKA may compromise in some patients with single-compartment osteoarthritis and under 65 years due to the higher level of activity. At the same time, initial TKA can be complicated in patients with severe varus deformities and it is important to choose a knowledgeable and experienced surgeon. Hence, primary HTO and secondary joint replacement can reduce the difficulty of operation and prolong the prosthesis survival rate. The purpose of this study is to quantify changes in radiological features and clinical outcomes in patients who were older than 60 years and underwent OWHTO.

## Patients and methods

After approved by the Ethics Committee of our hospital, patients who diagnosed with degenerative varus KOA and underwent OWHTO from August 2018 to August 2021 were retrospectively reviewed and analyzed. Patients were divided into two groups according the age; group G (Geriatric, ≥ 60 years) and group Y (Young, < 60 years). Demographic information and clinical or radiological data were extracted from the electronic medical record system (EMR) and picture archiving and communication system (PACs) by two well-trained investigators.

The inclusion criteria were: (i), patients who underwent primal OWHTO surgery; (ii), Body mass index (BMI) of patients was less than 30 kg/m^2^; (iii), radiographic signs of OA up to Ahlback grade II were tolerated; (iv), range of motion (ROM) of the knee larger than 90°; (v), MRI, stress x-ray film, patellar axial view and Rosenberg photograph were performed to insure the structural or functional integrity of anterior cruciate ligament (ACL), posterior cruciate ligament (PCL), medial collateral ligament (MCL) and lateral collateral ligament (LCL) and ideal cartilaginous state of the remaining compartments; (vi), extra-articular deformity larger than 5°, namely medial proximal tibial angle (MPTA) < 85°; (vii) patients with exclusive pain in the medial compartment.

The exclusion criteria were: (i), definite radiographic changes and clinical symptoms of lateral compartment or patellofemoral joint; (ii), OWHTO combine with distal femur osteotomy (DFO); (iii), rheumatoid arthritis and other inflammatory joint diseases; (iv), patients with a minimum follow-up time less than 12 months; (v), patients with an incomplete radiographic and other interested data.

### Preoperative planning and surgical procedure

Low extremity weight-bearing full-length photographs were obtained preoperatively, the weight-bearing axis was planned to pass the area between apex of tibial intercondylar eminence and center of knee joint, and weight-bearing line ratio (WBLR) was 50–55%. Miniaci method and Hernigou’s trigonometric chart were introduced to calculate the osteotomy gap opening depends (mm) on the mediolateral width of the osteotomy (mm) of the planned correction angle.

The OWHTO was performed by three senior surgeons, arthroscopic debridement was performed for each patient to directly evaluate the status of the articular cartilage and meniscus. Subsequently, a longitudinal incision was made on the 1/3 ~ 1/2 of the anteromedial aspect of the knee and part of the superficial MCL was retract by hooks, two Kirschner wire were inserted in the oblique direction from 3.5 cm below tibial plateau of medial proximal tibial cortex and located superior margin of pes anserinus, and was directed toward the lateral proximal tibial cortex at the level of the tip of the fibular head under fluoroscopic guidance. Horizontal osteotomy was performed beneath the wire using an osteotome and oscillating saw and osteotomies ended 10 mm medial to the lateral cortex. An ascending cut was made behind 1.5 cm posterior to the anterior rim of the tibial tuberosity and reserving 110° intersection with horizontal osteotomy, meanwhile, leaving the patellar ligament attached to the distal tibial fragment. Osteotomy gap was gradually opened and maintained by osteotomes and spreader, then force line pole was placed in the center of hip and ankle joint respectively and located at 50–55% of WBLR. TomoFix locking plate was used for internal fixation, and triangular tricortical iliac bone graft harvested from the ipsilateral iliac crest was required when the gap was larger than 13 mm.

Passive activities of the knee joint and partial weight-bearing (10 kg) were encouraged to carried out the day after surgery. Postoperative full ROM was granted immediately and crutches were also used to assist in walking. Anteroposterior and lateral X-ray images of knee joint and low extremity weight-bearing full-length photographs were taken at 3 days, 3 months, 6 months and 1 year after operation, full weight-bearing was allowed at 3 months after operation, if complete bone union were observed on the osteotomy gap and if radiographic healing is not achieved, complete non-weight bearing would extend.

### Clinical and radiographic outcomes

The clinical outcome of OWHTO were compared and analyzed using pre- and post-operative American Knee Score (AKS), visual analogue scale (VAS), Western Ontario and McMaster Universities Arthritis Index (WOMAC); moreover, physical examination was conducted to assessing the subjective presence or absence of lateral knee and PFJ pain. Radiographic evaluation of lower limb alignment was done using a weight-bearing full-length photographs by measuring WBLR, MPTA and posterior tibial slope angle (PTS) were also calculating on the antero-posterior and lateral radiograph. Meantime, radiographic osteoarthritic progressions of medical/lateral compartment and patellofemoral joints were determined using the Kellgren–Lawrence (K–L) grade.

### Statistical analysis

Statistical procedures were performed by SPSS 19.0 software package (SPSS Inc., Chicago, IL, USA). Kolmogorov–Smirnov test was performed firstly to determine whether the data conform to normal distribution. The continuous data were expressed as mean ± SD, whitney U-test was used for non-normally distributed continuous variables. The paired-samples t-test was used to determine the significance of change of mean AKS, ROM, WBLR, MPTA and PTS at preoperative and final follow-up period. Postoperative K–L grade was compared by the Chi square (χ^2^) test, and* P* value of 0.05 was considered to be statistically significant for all tests.

### Ethics approval

This study was performed in line with the principles of the Declaration of Helsinki. Approval was granted by the Ethics Committee of the First Hospital of Hebei Medical University.

### Consent to participate

Informed consent was obtained from all individual participants included in the study.

## Results

The patient demographic and baseline characteristics are summarized in Table 1. There were 18 male and 58 female patients in the present study with an F/M ratio of 3.2:1, the mean age of all the recruited patients was 58.5 ± 9.2 years (ranges from 40 to 82 years) and 51.4 ± 4.1 and 67.3 ± 4.9 years for group Y and G respectively. Thirty-five patients with one major comorbidity such as hypertension, coronary heart disease, diabetes mellitus, chronic obstructive pulmonary disease and so on, and 8 patients with more than two type of comorbidities, and 33 patients with no serious comorbidities. BMI for the 76 patients was 26.6 ± 3.2 kg/m^2^ and according to the Chinese criteria, there were 21.1%, 44.7%, and 27.6% patients with a normal (18.5–23.9 kg/m^2^), overweight (24–27.9 kg/m^2^) and obesity (28–31.9 kg/m^2^) BMI. Moreover, there were no significant differences in gender, BMI, and disease duration between the two group and more patients underwent bilateral OWHTO in younger patients (28.6 vs. 8.8%) and geriatric patients were more likely accompanied by one or more comorbidities (70.6 vs. 45.2%).

**Table 1:** Patients’ demographics and baseline characteristics.

Variables	Group G (n = 34)	Group Y (n = 42)	P
Age	67.3 ± 4.9	51.4 ± 4.1	0.000^†^
Gender (F/M)	6/28	12/30	0.265
BMI	26.0 ± 2.7	27.0 ± 3.4	0.159
Disease duration (year)	5.1 ± 3.9	4.6 ± 4.1	0.625
Surgical side (left/right/bilateral)	17/14/3	11/19/12	0.036^†^
Comorbidities (hypertension/CHD/diabetes mellitus)			
0	10	23	0.041^†^
1	18	17	
≥ 2	6	2	

*BMI* body mass index, *CHD* coronary heart disease.

^†^P < 0.05, significant variables.

There were 34 patients and 42 patients in group G and group Y respectively, Table 2 shows the preoperative radiological data and knee functional status between the two groups. There were 30 (71.4%) and 27 (79.4%) patients graded K–L II or III in group Y and G respectively, and no significant difference in MPTA, WBLR, PTS, and WOMAC, VAS, AKS, and ROM between the two group were found (P > 0.05).

**Table 2:** Comparison of preoperative radiological data and knee functional score between the two groups.

Variables	Group G (n = 34)	Group Y (n = 42)	P
K–L grade (≥ II)	27	30	0.424
MPTA (°)	80.8 ± 1.1	80.2 ± 2.1	0.165
WBLR (%)	21.0 ± 1.5	20.7 ± 1.6	0.454
PTS (°)	11.4 ± 1.1	11.2 ± 0.9	0.321
AKS	57.4 ± 6.4	52.0 ± 7.4	0.001†
WOMAC	104.5 ± 4.2	102.4 ± 16.6	0.474
VAS	7.0 ± 0.8	6.6 ± 1.0	0.053

*K–L* Kellgren–Lawrence grade, *MPTA* medial proximal tibial angle, *WBLR* weight-bearing line ratio, *PTS* posterior tibial slope angle, *AKS* American knee score, *WOMAC* Western Ontario and McMaster Universities Arthritis Index, *VAS* visual analogue scale.

^†^P < 0.05, significant variables.

After more than a mean two-year follow-up period, all patients can achieve a satisfactory clinical outcome. From Table 3 we can see that, postoperative WBLR, AKS, WOMAC, and VAS were much more desired than preoperative, PTS and ROM of the surgical knee were also similar to the preoperative ones, and more importantly, there was no significant difference in these variables between young and geriatric group (P > 0.05). As for the postoperative incidence of complications, geriatric patients are often accompanied by a delayed union when compared to the younger OWHTO patients (6.9% vs 4.7%). Meantime, radiographic progression of medical/lateral compartment and patellofemoral joints were determined using the Kellgren–Lawrence (K–L) grade. No significant postoperative radiographic osteoarthritic progressions of the remaining compartments based on K–L grade were observed when compared with the preoperative radiologic data.

**Table 3:** Comparison of postoperative variables and complications between patients underwent OWHTO.

Variables	Group G (n = 34)	Group Y (n = 42)	P
WBLR (%)	54.2 ± 1.5	53.9 ± 1.5	0.430
PTS (°)	8.5 ± 0.5	8.4 ± 0.5	0.295
AKS	85.4 ± 4.2	86.2 ± 4.0	0.390
WOMAC	47.5 ± 5.8	48.1 ± 7.1	0.717
VAS	1.9 ± 1.6	1.6 ± 0.7	0.261
Complication			
SSI	0	0	n.s
DVT	3	7	0.315
Delayed union	3	2	0.478

*SSI* surgical site infection, *DVT* deep venous thrombosis.

^†^P < 0.05, significant variables.

## Discussion

OWHTO is an established operation in the treatment of medical compartment osteoarthritis, and characterized by extra-articular procedure, which can redistribute the alignment of the lower extremity, therefore, cartilage of the remaining compartments can be reserved and better postoperative ROM can be obtained. Currently, HTO is generally performed in patients younger than 60 years^7^. However, some scholars have demonstrated the good outcome of HTO in elderly patients with age up to 72 years^8^. In this present study, 76 patients have undergone OWHTO with a mean age of 58.5 years with minimum age of 40, and a maximum age of 82, and we found that short-term postoperative clinical outcomes and functional status of knee joint for the geriatric patients (older than 60 years) were comparable to the younger HTOs.

The biomechanical and physiological mechanisms of HTO can be summarized as follows: (i), a normal biomechanical environment of the knee joint was restored by correcting the varus deformity and alignment, and when the abnormally high pressure of the medial compartment is relieved and released, the favorable biochemical environment of the medial compartment is conducive to the repair of worn articular cartilage; (ii), intraosseous pressure decreased, and venous stasis anesis acquired after OWHTO, therefore, local hemodynamic and metabolic of the medical femoral condyle and tibial plateau were changed, meanwhile, the incidence of microfractures under the medical tibial plateau decreased; (iii), the injured articular cartilage can be repaired and new hyaline cartilage is formed; degeneration of cartilage would also be delayed, subsequently the clinical symptoms and signs can be eliminated or relieved.

Although most authors recommend the most ideal age distribution for HTO was 40–60 years^9^, however, with the improvement of implant design, surgical methods, and application of enhanced recovery after surgery (ERAS), indication of OWHTO was expanded especially in term of patients’ age^10^. Lee et al. have conducted an investigation and evaluated the influence of age on survivorship and complications after HTO, there were 41,112, 13,895 and 6138 patients underwent HTO under 60 years (Group A), between 60 and 65 years (Group B) and older than 65 years (Group C), results showed that overall revision rate was 4.2%, 6.4% and 7.3% for Group A, B, C. The 5- and 10-year revision rate and incidence of complication were significantly lower in Group A^11^. However, other studies also reported good outcomes of HTO in geriatric patients, Hrishikesh and his colleagues analyzed the functional changes of varus degenerative OA knee by hemicallotasis using modular dynamic HTO fixator, mean age of the study population in their research was 49.3 years with a maximum of 67 years, the KOOS improved from mean 56.61 preoperatively to 70.48 at the time of fixator removal, and after 1-year follow-up, KOOS score further improved significantly to 85.68^12^. It is well accepted that a mildly degenerative KOA is usually accompanied by better clinical outcomes of HTO, therefore, we performed OWHTO in patients with K–L grade up to III, and 77.6% of the OWHTO patients in our study were assessed as grade I or II of K–L grade. Forty-two (55.2%) patients younger than 60 years, 26 (34.2%) patients aged 60–70 years, eight (10.5%) patients aged > 70 years old in the present study. After more than 2-year follow-up, we found AKS and WOMAC scores for the geriatric group were comparable to the younger ones, however, the incidence of postoperative complications was higher for geriatric patients and this result was consistent with current research findings^11^.

Traditionally, surgical treatment of KOA could be osteochondral transplantation, arthroscopic debridement, osteotomy around the knee and joint replacement, and TKA sometimes involves removing healthy joint surfaces^13^. Recent research has looked into the potential benefits of options for knee preservation such as UKA and HTO. It should be pointed out that although we have observed satisfactory clinical results in elderly patients undergoing HTO in our study, the important role of joint replacement in treatment of KOA has been widely proven. UKA is an established surgical treatment for isolated knee osteoarthritis OA with the absence of significant coronal deformity or flexion contracture. From global perspective, the usage of UKA is constantly increased over the decade, although, in short-term outcomes, TKA had higher postoperative complications than UKA, one disadvantage for UKA is the higher revision rate^14^, and Murray DW^15^ recommend that the best way to minimize the revision rate is for surgeons to use UKA for at least 20% of their knee arthroplasties. Personalized prostheses also play an important role in TKA, Benazzo F and his colleagues^16^ designed an anatomically tibial component implant with a proportional medialized tibial keel, and patients defined surgical results as excellent in 66%, very good in 23%, good in 5% of the cases and the results showed an excellent outcome of this design.

In this study, the better clinical outcomes of OWHTO in the elderly population are mainly attributed to the relatively strict radiologic selection and physical examination, the ideal histological condition of cartilage in femoral patellar joint and lateral compartment reduces the risk of postoperative anterior and lateral knee pain, moreover, the lower K–L grade further decelerate the joint degeneration.

## Strengths and limitations

The criteria of our study ensure the HTO treatment prognosis, however, it also leads to a small sample number of eligible patients, especially geriatric ones, this is the main limitation of our research. What is more, survivorship and revision rate which scholars are more concerned about, could not be evaluated objectively due to the short follow-up time for the present study, and this is one of the objectives that we should continue to explore in future.

## Conclusion

OWHTO plays an important role in the treatment of KOA and can avoid geriatric patients from undergoing secondary knee surgery in the short term; elderly patients receiving OWHTO should be under strict indication and long-term follow-up outcomes need to be widely confirmed by different researchers to ultimately clarify the survival rate of OWHTO in geriatric patients.

## Data Availability

The datasets generated and/or analysed during the current study are not publicly available due to protection of patients' privacy but are available from the corresponding author on reasonable request.

## Abbreviations

KOA: Knee osteoarthritis
HTO: High tibial osteotomy
CWHTO: Closing wedge HTO
OWHTO: Opening wedge HTO
TKA: Total knee arthroplasty
UKA: Unicompartmental knee arthroplasty
EMR: Electronic medical record system
PACs: Picture archiving and communication system
ROM: Range of motion
ACL: Anterior cruciate ligament
PCL: Posterior cruciate ligament
MCL: Medial collateral ligament
LCL: Lateral collateral ligament
MPTA: Medial proximal tibial angle
K–L: Kellgren–Lawrence grade
WBLR: Weight-bearing line ratio
PTS: Posterior tibial slope angle
AKS: American knee score
WOMAC: Western Ontario and McMaster Universities arthritis index
VAS: Visual analogue scale
DFO: Distal femur osteotomy
